# Biomechanical, biochemical, and near infrared spectral data of bovine knee ligaments and patellar tendon

**DOI:** 10.1016/j.dib.2021.106976

**Published:** 2021-03-19

**Authors:** Aapo Ristaniemi, Jari Torniainen, Tommi Paakkonen, Lauri Stenroth, Mikko A.J. Finnilä, Petri Tanska, Juha Töyräs, Rami K. Korhonen

**Affiliations:** a Department of Applied Physics, University of Eastern Finland, Kuopio, Finland; b Diagnostic Imaging Center, Kuopio University Hospital, Kuopio, Finland; c School of Medicine, University of Eastern Finland, Kuopio, Finland; d Research Unit of Medical Imaging, Physics and Technology, University of Oulu, Oulu, Finland; e School of Information Technology and Electrical Engineering, The University of Queensland, Brisbane, Australia

**Keywords:** Knee ligaments, Patellar tendon, Biomechanics, Biochemistry, Near infrared spectroscopy

## Abstract

Knee joint ligaments and patellar tendon are rope-like tissues that enable the proper function of the knee by connecting the bones that form the joint. A better understanding of ligament structure-function relationships is needed to develop objective and reliable diagnostic methods for ligaments. Recently, arthroscopic near infrared spectroscopy (NIR) has shown the potential to quantitatively evaluate the health of the cartilages and menisci of the knee. In this dataset, we present a unique combination of NIR spectral data, biomechanical properties, and biochemical composition of bovine primary knee ligaments and patellar tendon (10 knees, 50 tissue samples). NIR spectral data were measured at 5 locations in each sample, biomechanical properties were obtained with tensile testing, and biochemical composition was quantified using colorimetric biochemical methods. The data can be reused for investigations of structure-function relationships of knee ligaments and patellar tendon, for the development of NIR spectroscopic methods to quantify the health of these tissues, and to develop new computational models to describe ligament and tendon biomechanics.

## Specifications Table

**Table:** 

Subject	Biophysics
Specific subject area	Mechanical, chemical and near infrared spectral characterization of knee ligaments and patellar tendon
Type of data	Table
How data were acquired	Near Infrared Spectrometer (AvaSpec Multichannel ULS2048L-USB2/NIR256–2.5-HSC Spectrometer, Avantes BV, Apeldoorn, Netherlands) and a 10 W Tungsten Halogen light source. Uniaxial material testing system with a linear actuator (Newport, Irvine, CA, USA), controller (Newport, Irvine, CA, USA), and 25 lb load cell (Model 31/AL311BL, Honeywell, Columbus, OH, USA). Measurements controlled with LabView (version 10.0, National Instruments Corporation, Austin, TX, USA). Data stored with Microsoft Excel (Microsoft Corporation, Redmond, WA, USA) and comma separated tables. Data analysed with MATLAB (MathWorks Inc., Natick, MA, USA).
Data format	Raw Analyzed
Parameters for data collection	Ten bovine stifle joints (anatomically corresponding to the human knee joint), aged 14–22 months, were collected at an abattoir (Atria Oyj, Seinäjoki, Finland). Samples were extracted from the anterior cruciate, posterior cruciate, medial collateral, and lateral collateral ligaments and from the patellar tendon.
Description of data collection	Collected samples were subjected to (in this order) spectral, mechanical and chemical characterization.
Data source location	Department of Applied Physics University of Eastern Finland Kuopio, Finland
Data accessibility	Repository name: Fairdata Research Data Storage Service Data identification number: 10.23729/40c4f149-d894–4126-bad2–79fe47b3e2fa Direct URL to data: https://etsin.fairdata.fi/dataset/925999ad-8127–4428-b7cc-c03c12e62d3c/data
Related research article	Ristaniemi, A., Stenroth, L., Mikkonen, S. & Korhonen, R. K. Comparison of elastic, viscoelastic and failure tensile material properties of knee ligaments and patellar tendon. *J. Biomech.***79**, 31–38 (2018).

## Value of the Data

• This dataset is useful in ligament and tendon basic research, as their structure-function relationships are not yet fully understood.
• The dataset is intended for researchers investigating the biomechanical behavior and biochemistry of ligaments and tendons. The included NIRS measurements are useful for biophotonics researchers interested in quantitative evaluation of connective tissues.
• Biomechanical data can be used in the development of new computational models describing their mechanics. NIRS data can be utilized to develop multivariate models for quantitative evaluation of various ligament properties.

## 1 Data Description

The data are stored in several Microsoft Excel (Microsoft Corporation, Redmond, WA, USA) files, stored in *Fairdata Research Data Storage Service* [1]. Alternatively, the same data are given in comma separated value (.csv) files. Table 1 presents an overview of the methods, number of measurements and generated data.

**Table 1:** Summary of the measurement methods, number of measurements, and the corresponding data.

Method	Number of measurements	Data
Near infrared spectroscopy	250	Absorption spectra
Biomechanical testing (tensile testing)	50	Raw data (time, displacement, load) and analyzed parameters
Freeze-drying	50	Water content (water mass fraction)
Colorimetric biochemical method^11^	50	Hydroxyproline content per wet and dry weights
Colorimetric biochemical method^12^	50	Uronic acid content per wet and dry weights
Colorimetric biochemical method^13^	50	Elastin content per wet and dry weights

### 1.1 Near infrared spectral data

Near infrared spectral data are stored in an Excel file *Data_NIRS.xlsx*, which contains the measured spectrum of each sample. Example codes *nirs_plsr_analysis.m and nirs_visualize_spectra.m* showing good practices on preprocessing, analysis and visualization of NIR spectral data were created with MATLAB R2016b (Mathworks Inc., Natick, MA, USA) and it is available publicly (https://github.com/UEF-BBC/ristaniemi-data-in-brief-2021).

**File 1.** Data_NIRS.xlsx
SHEET – AvaSpec-ULS2048L-USB2 (name of the detector used for wavelengths 320–1100 nm)
COLUMN A – Sample name (M01_ACL, M02_ACL, etc.) where M01…M10 denotes the knee number and ACL, LCL, MCL, PCL or PT the tissue type
COLUMN B – Site of the measurement, 1–5, where 1 is the measurement at the proximal and 5 at the distal end of the sample.
COLUMNS C-AYS – Absorption value at different wavelengths, wavelength shown in the first row.
SHEET – AvaSpec-NIR256–2.5-HSC (name of the detector used for wavelengths 950–2500 nm)
COLUMN A – Sample name (M01_ACL, M02_ACL, etc.) where M01…M10 denotes the knee number and ACL, LCL, MCL, PCL or PT the tissue type
COLUMN B – Site of the measurement, 1–5, where 1 is the measurement at the proximal and 5 at the distal end of the sample.
COLUMNS C-IK – Absorption value at different wavelengths, wavelength shown in the first row.

### 1.2 Biomechanical and biochemical data

Biomechanical and biochemical data are stored in an Excel table *Data_biomechanics_and_biochemistry.xlsx* containing the properties (e.g. Young's modulus, hydroxyproline content, etc.) of each sample measured.

**File 2.** Data_biomechanics_and_biochemistry.xlsx
SHEET – Data
COLUMN A – Sample name (M1_ACL, M2_ACL, etc.) where M1…M10 denotes the knee number and ACL, LCL, MCL, PCL or PT the tissue type
COLUMN B – Knee identifier
COLUMN C – Tissue type
COLUMN D – Phase difference at 0.1 Hz loading
COLUMN E – Phase difference at 0.5 Hz loading
COLUMN F – Phase difference at 1 Hz loading
COLUMN G – Dynamic modulus at 0.1 Hz loading
COLUMN H – Dynamic modulus at 0.5 Hz loading
COLUMN I – Dynamic modulus at 1 Hz loading
COLUMN J – Young's modulus
COLUMN K – Toe region fit coefficient A
COLUMN L – Toe region fit coefficient B
COLUMN M – Toe region fit coefficient C
COLUMN N – Toe region fit coefficient D
COLUMN O – Toe region fit coefficient F
COLUMN P – Linear region length
COLUMN Q – Toe region strain
COLUMN R – Toe region stress
COLUMN S – Yield strain
COLUMN T – Yield stress
COLUMN U – Toughness at yield
COLUMN V – Ultimate strain
COLUMN W – Ultimate strength
COLUMN X – Toughness at failure
COLUMN Y – Water content
COLUMN Z – Hydroxyproline content of wet weight
COLUMN AA – Hydroxyproline content of dry weight
COLUMN AB – Uronic acid content of wet weight
COLUMN AC – Uronic acid content of dry weight
COLUMN AD – Elastin content of wet weight
COLUMN AE – Elastin content of dry weight

Additionally, the raw data of the tensile tests are provided (Files 3–52) to enable the reuse of the experimental biomechanical measurements. For each sample, there is an Excel file containing the time, upper clamp displacement, and load signals of the relaxation, sinusoidal and ultimate tests. Moreover, one sheet in that excel file indicates the zero-load length, thickness, and width in millimeters. An example code *Biomechanical_analysis_example.m* to calculate the mechanical properties from the raw data was created with MATLAB R2018B, and it is available publicly (https://github.com/UEF-BBC/ristaniemi-data-in-brief-2021).

**Files 3–52.** M1_ACL.xlsx, M2_ACL.xlsx, etc. (one file for each sample)
SHEET – M1_ACL_relax (sheet includes the sample name)
COLUMN A – Time of the relaxation test in seconds
COLUMN B – Displacement of the upper clamp during the relaxation test in micrometers
COLUMN C – Load signal of the relaxation test in grams
SHEET – M1_ACL_sin1 (sheet includes the sample name)
COLUMN A – Time increments of the sinusoidal test at 0.1 Hz in seconds
COLUMN B – Displacement of the upper clamp during the sinusoidal test at 0.1 Hz in micrometers
COLUMN C – Load signal of the sinusoidal test at 0.1 Hz in grams
SHEET – M1_ACL_sin2 (sheet includes the sample name)
COLUMN A – Time increments of the sinusoidal test at 0.5 Hz in seconds
COLUMN B – Displacement of the upper clamp during the sinusoidal test at 0.5 Hz in micrometers
COLUMN C – Load signal of the sinusoidal test at 0.5 Hz in grams
SHEET – M1_ACL_sin3 (sheet includes the sample name)
COLUMN A – Time increments of the sinusoidal test at 1 Hz in seconds
COLUMN B – Displacement of the upper clamp during the sinusoidal test at 1 Hz in micrometers
COLUMN C – Load signal of the sinusoidal test at 1 Hz in grams
SHEET – M1_ACL_UTS (sheet includes the sample name)
COLUMN A – Time of the ultimate test in seconds
COLUMN B – Displacement of the upper clamp during the ultimate test in micrometers
COLUMN C – Load signal of the ultimate test in grams
SHEET – M1_ACL_l_t_w (sheet includes the sample name)
COLUMN A – Length of the sample in millimeters
COLUMN B – Thickness of the sample in millimeters
COLUMN C – Width of the sample in millimeters

## 2 Experimental Design, Materials and Methods

### 2.1 Sample preparation

**Fig. 1:**
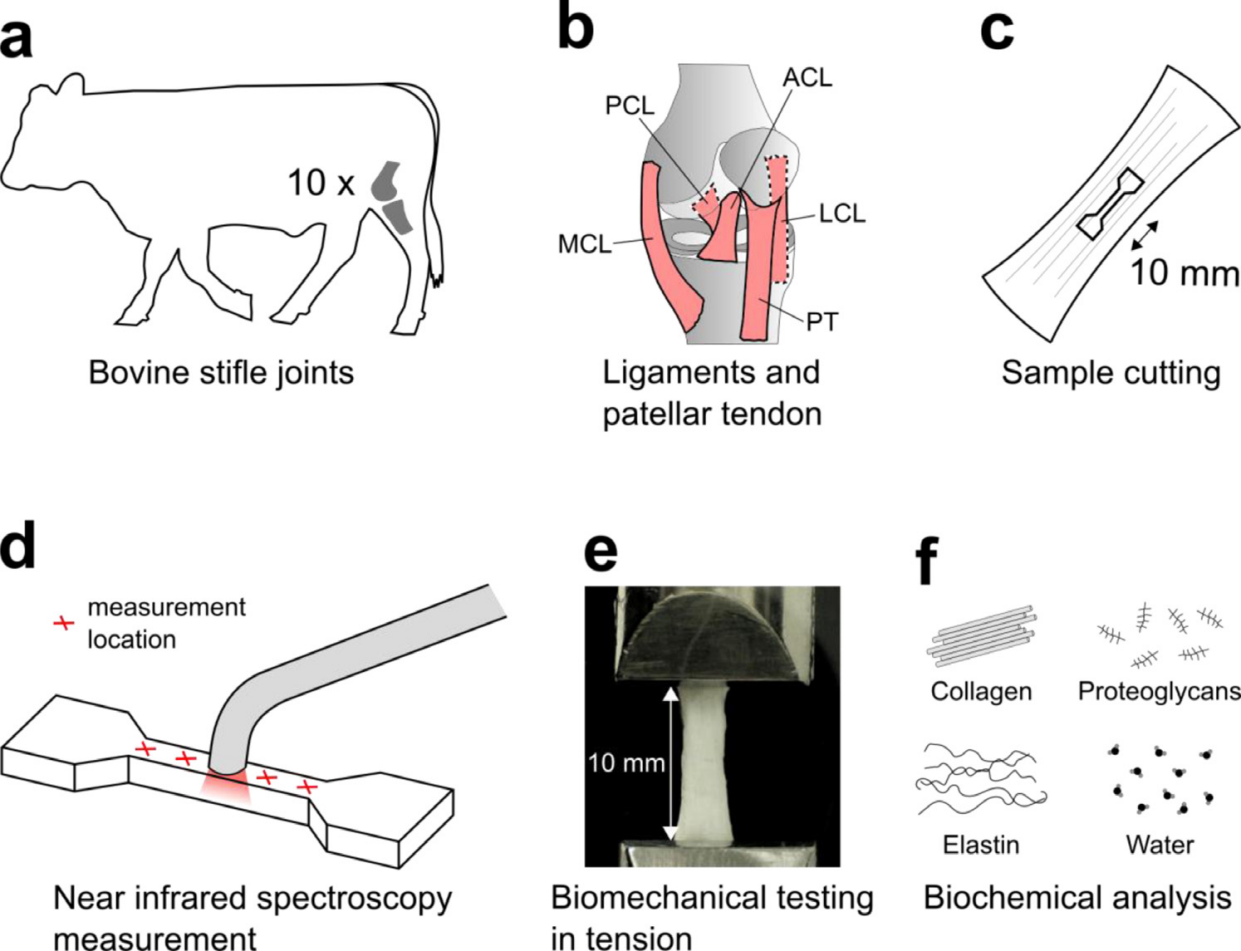
Overview of the data collection protocol. **a**) Bovine stifle joints used in the study. **b**) Primary ligaments and PT. **c**) Dogbone-shaped sample preparation. **d**) Near infrared spectroscopy measurement locations. **e**) Biomechanical tensile testing. **f**) Analysis of biochemical composition.

Ten bovine stifle joints (anatomically corresponding to the human knee joint), aged 14–22 months, were collected at an abattoir (Atria Oyj, Seinäjoki, Finland) (Fig. 1a). The ACL, PCL, MCL, LCL, and PT were carefully extracted, placed in plastic containers filled with phosphate-buffered saline solution (PBS), and put in a freezer (−20 °C) (Fig. 1b). The PBS contained two enzyme-inhibitors to enhance sample preservation, the ethylenediaminetetraacetic acid disodium salt (1.86 g/L, EDTA VWR International, Radnor, PA, USA) and benzamidine hydrochloride hydrate (0.78 g/L, Sigma-Aldrich Co., St. Louis, MO, USA). Prior to the measurements, the samples were thawed at room temperature (approximately 21 °C), and a dogbone-shaped test piece was cut from the ligament mid-substance, with collagen fibers directed to the longitudinal axis (Fig. 1c). First, two parallel razor blades, fixed to a separation of 1.81 mm, were used to cut a slice from the tissue. Then, the desired dogbone-shape was cut from the slice using a custom punch tool. The central part of the dogbone shape had a width of approximately 2 mm, and a length of 10 mm. This type of shape and size has been used earlier when studying mechanical properties of ligaments and tendons [2], [3], [4], [5], [6], [7].

### 2.2 Near infrared spectroscopy

**Fig. 2:**
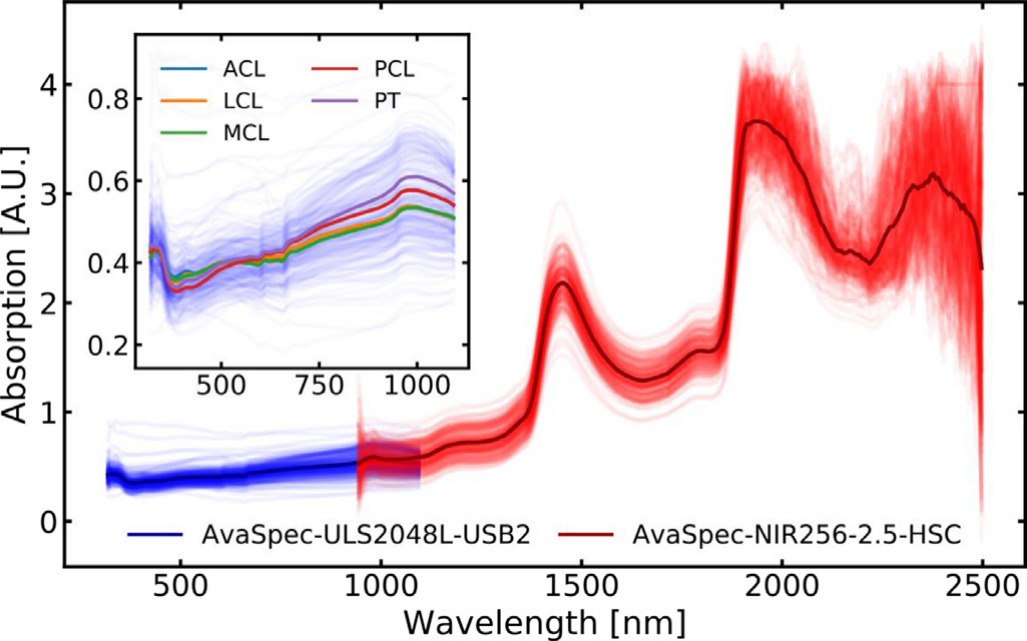
Near infrared spectroscopic measurements (*N* = 250) from the two detectors. Thin lines correspond to individual measurements while thick lines represent the average spectrum. The subfigure illustrates the spectral differences between the ligament and tendon types in the spectral range of 320–1100 nm.

NIRS measurements were conducted using a dual-detector NIRS system (AvaSpec Multichannel Spectrometer, Avantes BV, Apeldoorn, Netherlands) with a 10 W Tungsten Halogen light source. The two detectors of the system covered wavelength regions of 320–1100 nm (AvaSpec-ULS2048L-USB2, resolution 0.6 nm) and 950–2500 nm (AvaSpec-NIR256–2.5-HSC, resolution 3.2 nm), respectively (Fig. 2). The system was connected to a custom stainless-steel probe shaped like an arthroscopic hook (outer diameter=3.25 mm, inner diameter=1.90 mm). The probe contained a total of 114 optical fibers (fiber diameter=100 µm); 100 fibers for emitting and 14 fibers for collecting light to the spectrometers (i.e., seven fibers per detector). NIR spectra were acquired with 100 coadded scans using an integration time of 1.5 milliseconds for AvaSpec-ULS2048L-USB2 detector and 20 milliseconds for AvaSpec-NIR256–2.5-HSC detector. Measurements were taken from five equispaced and non-overlapping sites (4.0 mm spacing between measurement center points) along the longitudinal axis of the sample (250 NIR spectra in total, Table 1).

Prior to each measurement, the system was calibrated with a diffuse reflectance standard (99% ± 4% reflectance factor in the 200–2500 nm range, Spectralon SRS-99, Labsphere Inc., North Sutton, USA) and a non-reflective piece of black rubber. The non-reflective rubber corresponded to the background noise in the absence of any incoming light, while the reflectance standard represents the maximum signal-to-noise ratio the detector can reach without saturating the signal. Absorption values (A) for each individual wavelength (λ) were computed with the following equation,

(1) $$A\left(\lambda \right)=-\log_{10}\frac{S\left(\lambda \right)-D\left(\lambda \right)}{R\left(\lambda \right)-D\left(\lambda \right)}$$

where S(λ) is the measured spectrum, D(λ) the spectrum of the non-reflective standard, and R(λ) the spectrum of the reflectance standard.

### 2.3 Biomechanical testing

The width and thickness at the sample center cross-section were measured with an optical microscope using 4.6x magnification, and elliptical shape was assumed to determine the cross-sectional area [8], [9], [10]. The elliptical assumption was verified to be accurate, with a 0.15% error in the resulting cross-sectional area compared with a direct measurement of a cut cross-section, using the same microscope and magnification [11]. Double-sided sandpapers (Mirox P80, Mirka Oy, Uusikaarlepyy, Finland) were glued (Loctite Precision, Henkel AG, Düsseldorf, Germany) to both sides at both ends of the sample to ensure a good grip in tensile testing. The sample was carefully placed between jaw-type clamps and aligned with the machine axis, and the clamp screws were adjusted to a 4 Nm moment with a torque wrench. This procedure ensured constant and proper clamping for all the samples, and no slipping was observed during the tests. The samples were kept at room temperature (approximately 21 °C) in PBS during the tensile testing. At body temperature (37 °C) the samples may exhibit unaltered quasi-static behavior [12], but altered history-dependent viscoelastic properties [13].

**Fig. 3:**
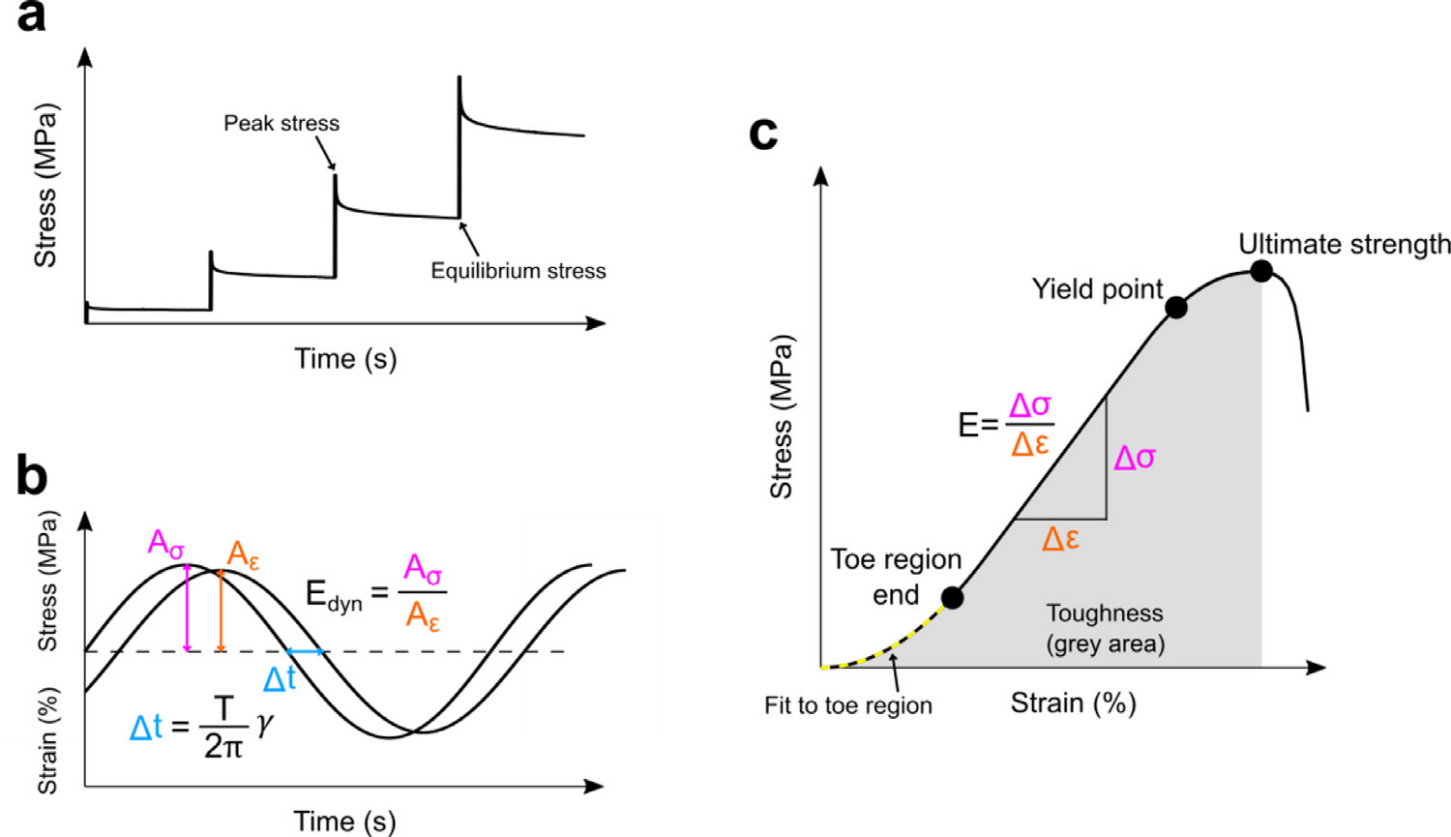
Biomechanical testing methods and parameters. (**a**) Four-step stress-relaxation test. (**b**) Sinusoidal loading test. (**c**) Tensile test until tissue failure. $A_{\sigma }$=stress amplitude, $A_{\varepsilon }$=strain amplitude,$\;E_{dyn}$=dynamic modulus, Δt=time difference, *T*=period, γ=phase difference, *E*=Young's modulus, Δσ=change in stress, Δε=change in strain.

A uniaxial material testing system was used to conduct the tensile testing. It consisted of a linear actuator (Newport, Irvine, CA, USA; resolution 0.1 µm) and a controller (Newport, Irvine, CA, USA), operated via a custom LabView software (version 10.0, National Instruments Corporation, Austin, TX, USA), and a 25 lb load cell (Model 31/AL311BL, Honeywell, Columbus, OH, USA). Tensile stress of 0.05 MPa was applied to determine the zero-load length [3]. The preconditioning protocol consisted of 10 loading-unloading cycles with 0.05 mm/s velocity to 2% strain. A 2-min recovery period was allowed, and the zero-load length was re-established. This 10-cycle protocol was repeated five times to obtain a stable mechanical behavior [11]. Then, a 10-min recovery was allowed, the zero-load length was verified and the measurement was started. An incremental stress-relaxation test was conducted at 2, 4, 6 and 8% strain, with ramp speed of 0.1 mm/s (i.e. ~1%/s), and 30 min of relaxation at each strain level, exhibiting relaxation behavior useful for viscoelastic material model development (Fig. 3a). After the relaxation, with the sample at 8% strain, a sinusoidal test was imposed with 0.5% strain amplitude [14] with 0.1, 0.5 and 1 Hz frequencies for 20 cycles at each frequency (Fig. 3b). The sample was then allowed to recover at 0% strain (zero-load length) for 1 h. Finally, a tensile test until failure was performed with 0.005 mm/s (0.05%/s) velocity (Fig. 3c).

To calculate the material parameters, the sinusoidal and ultimate test force and displacement data were used to calculate the true stress ($\sigma =\frac{F}{A_{0}}\frac{L}{L_{0}}$, where F is the measured force, $A_{0}$ the initial cross-sectional area, L the current length and $L_{0}$ the initial length) and logarithmic strain ($\varepsilon =\ln\frac{L}{L_{0}}$). The true stress assumes a constant volume of the material. For the sinusoidal test, a sinusoidal function was fit to stress-time and strain-time data:

(2) $$z=A\;\text{sin}\left(2\pi ft+\varphi \right)+z_{0},$$

where z is the stress or strain, A is the amplitude, f is the frequency, t is the time, φ is the phase angle and $z_{0}$ is the constant term. The dynamic modulus $E_{dyn}$ and phase difference γ were then calculated as

(3) $$E_{dyn}=\frac{A_{\sigma }}{A_{\varepsilon }},$$

and

(4) $$\gamma =\varphi_{\sigma }-\varphi_{\varepsilon },$$

where the subscripts σ and ε denote the parameters related to stress-time and strain-time data (Fig. 3b).

For the ultimate test, the data from 0.2 MPa onwards were taken for analysis, to exclude the slack portion from the analysis. To determine the Young's modulus, linear fits were made to the stress-strain curve and the fit with the maximum tangent modulus [15] was used to define the Young's modulus. More precisely, at each data point of the curve, the successive points covering an 8% strain interval were taken and a linear fit was made to determine the modulus. The maximum of these moduli, which practically occurs in the linear region, was then defined as the Young's modulus (Fig. 3c). Then, the toe region end ($\varepsilon_{toe}$,$\;\sigma_{toe}$) and yield points ($\varepsilon_{yield}$,$\;\sigma_{yield}$) were defined as points where the experimental stress-strain curve deviated by 0.6% strain [16] from the linear fit with the maximum modulus. The maximum stress reached during the experiment was defined as the ultimate strength ($\varepsilon_{ult}$,$\;\sigma_{ult}$). The linear region length was then calculated as $\varepsilon_{linear}=\varepsilon_{yield}-\varepsilon_{toe}$. Toughness K, or energy density, was calculated as the integral over the stress-strain curve to the ultimate strain. In addition, we calculated this parameter also at yield point:

(5) $$K_{yield/ult}=\int_{0}^{\varepsilon_{yield/ult}}\sigma \;d\varepsilon .$$

The toe region, $0<\varepsilon <\varepsilon_{toe}$, was characterized by a second order formula [16]

(6) $$\sigma =A\varepsilon^{2}+B\varepsilon +C,$$

where A, B and C are constants, and with an exponential formula [17]

(7) $$\sigma =D\left(e^{F\varepsilon }-1\right),$$

where D and F are constants.

### 2.4 Biochemical analyses

The sample surface was blot with a paper towel to remove surplus PBS, and the wet weight was taken as the average of three measurements. The samples were lyophilized (freeze-dried) for 24 h, and the dry weight was taken as the average of three measurements. Water content was subsequently determined by the difference between wet and dry weights. One part of the samples was used to determine the hydroxyproline and uronic acid contents. The samples were incubated in 150 mM sodium acetate including 5 mM l-cysteine, with 15 mM EDTA and a 1 mg/ml concentration of papain at pH of 5.8 and 60 °C for 16 h to digest the proteoglycans [18]. The samples were then boiled for 10 min for the deactivation of the enzyme. The hydroxyproline content was quantified following the procedure outlined in Brown et al. [19]. After papain digestion, the samples were hydrolyzed in 10 M HCl at 108 °C for 16 h. Following cooling, 1 M sodium hydroxide was used to neutralize the samples. An oxidizing solution with chloramine-T was added and mixed well, and allowed to react for 5 min. Ehrlich's reagent was then added to each sample and they were mixed using a vortex mixer. The samples were then incubated in a water bath at 60 °C for 45 min. A microplate reader was used to read the absorbance at 540 nm. The hydroxyproline content was determined three times and averaged, and the content was normalized by wet and dry weights.

The uronic acid content was quantified as outlined in Blumenkrantz and Asboe-Hansen [20]. First, ethanol precipitation was used to remove possible salts from the papain digested samples. Then, samples were cooled on crushed ice and sulfuric acid/sodium tetraborate solution was added. The samples were shaken and warmed in a water bath at 100 °C for 5 min. The samples were cooled, m-hydroxyphenyl reagent was added and after 5 min the absorbance was read at 540 nm. The uronic acid content was determined three times and averaged, and the content was normalized by wet and dry weights.

The elastin content was determined with Fastin Elastin Assay (Biocolor Ltd., Carrickfergus, County Antrim, United Kingdom) according to the manufacturer's instructions [21], using the other part of the samples. First, the samples were boiled in 0.25 M oxalic acid for 1 h to extract the elastin. To ensure that all elastin was recovered, boiling was repeated three times, which was verified to be sufficient in our pilot measurements. Elastin was precipitated with a precipitation reagent from two aliquots of pooled extracts, and reacted with dye for 90 min. The dyed elastin, red-brown in color, was dissociated and the solution was placed in a microwell plate. The absorbance was read at 513 nm and the elastin content was determined by comparison to a standard curve. The content was normalized by wet and dry weights.

## Ethics Statement

The tissues used in this study were obtained from an abattoir, as leftovers from meat production, therefore no further ethical approvals were required for this study.

## CRediT Author Statement

**Aapo Ristaniemi:** Investigation, Methodology, Formal analysis, Software, Visualization, Data curation, Writing - Original Draft; **Jari Torniainen:** Investigation, Methodology, Formal analysis, Software, Visualization, Data curation, Writing - Review & Editing; **Tommi Paakkonen:** Methodology, Investigation, Writing - Review & Editing; **Lauri Stenroth:** Conceptualization, Supervision, Writing - Review & Editing; **Mikko A. J. Finnilä:** Conceptualization, Supervision, Writing - Review & Editing; **Petri Tanska:** Conceptualization, Supervision, Writing - Review & Editing; **Juha Töyräs:** Conceptualization, Funding acquisition, Supervision, Resources, Project administration, Writing - Review & Editing; **Rami K. Korhonen:** Conceptualization, Funding acquisition, Supervision, Resources, Project administration, Writing - Review & Editing.

## Declaration of Competing Interest

The authors declare that they have no known competing financial interests or personal relationships which have or could be perceived to have influenced the work reported in this article.
